# Use of a Mobile App to Augment Psychotherapy in a Community Psychiatric Clinic: Feasibility and Fidelity Trial

**DOI:** 10.2196/17722

**Published:** 2020-07-03

**Authors:** Atif Adam, Ameena Jain, Alexandra Pletnikova, Rishi Bagga, Allison Vita, Lisa N Richey, Neda Gould, Supriya Munshaw, Kavi Misrilall, Matthew E Peters

**Affiliations:** 1 Rose: Smarter Mental Health Washington, DC United States; 2 Key Point Health Services, Inc Baltimore, MD United States; 3 Department of Psychiatry and Behavioral Sciences Johns Hopkins University Baltimore, MD United States; 4 Carey Business School Johns Hopkins University Baltimore, MD United States

**Keywords:** mobile app, mental health, depression, anxiety

## Abstract

**Background:**

Even though 1 in 5 Americans experience some form of mental illness each year, 80% have been shown to discontinue psychotherapy prematurely. The traditional psychotherapy service delivery model, consisting of isolated clinical sessions, lacks the ability to keep patients engaged outside clinical sessions. Newer digital mental health platforms can address the clinical need for a robust tool that tracks mental well-being and improves engagement in patients with depressive symptoms.

**Objective:**

The primary goals of this feasibility study were to (1) assess compliance among providers and their patients with a digital mental health platform protocol, and (2) examine the usability and fidelity of a mobile app through structured participant feedback.

**Methods:**

A sample of 30 participants was recruited for a 5-week study from a community-based mental health clinic in Baltimore, Maryland, USA. Inclusion criteria were: aged 18 years or older, having access to a smartphone, and having at least mild-to-moderate depression and/or anxiety as measured by the Patient Health Questionnaire-9 (PHQ-9) and Generalized Anxiety Disorder-7 (GAD-7) scales, respectively. Eligible participants were randomized into one of two study arms: (1) the intervention arm or (2) the waitlist control arm. Participants in the intervention arm were asked to download the Rose app and were prompted to complete clinical assessments (PHQ-9 and GAD-7) every other week, daily mood and anxiety Likert scales, and daily journal entries. The participants in the waitlist arm served as controls for the study and completed the clinical assessments only. Both arms engaged in weekly psychotherapy sessions, with participant in-app input informing the psychotherapy process of the intervention arm, while those in the waitlist control arm continued their standard care. Outcomes of interest included adherence to completion of in-app assessments and usability of the Rose mobile app assessed through the modified Mobile Application Rating Scale.

**Results:**

Over the study period, a sample of 30 participants used the Rose app 2834 times to complete clinical assessments. On average, 70% (21; 95% CI 61.14%-77.41%) of participants completed mood and anxiety daily check-ins and journal entries 5 days per week. Nearly all participants (29/30, 97%) completed all PHQ-9 and GAD-7 in-app scales during the study. Subjective impressions showed that 73% (22/30) of participants found the mobile app to be engaging and in line with their needs, and approximately 70% (21/30) of participants reported the app functionality and quality of information to be excellent. Additionally, more than two-thirds of the participants felt that their knowledge and awareness of depression and anxiety management improved through using the app.

**Conclusions:**

Steady compliance and high app ratings showcase the utility of the Rose mobile mental health app in augmenting the psychotherapy process for patients with mood disorders and improving mental health knowledge. Future studies are needed to further examine the impact of Rose on treatment outcomes.

**Trial Registration:**

ClinicalTrials.gov NCT04200170; https://clinicaltrials.gov/ct2/show/NCT04200170

## Introduction

Each year, 1 in 5 Americans (approximately 46.6 million people) experience some form of mental illness [1-3]. The National Institute of Mental Health estimated that 17.3 million adults in the United States had at least one major depressive episode in 2017, representing 7.1% of the population. However, studies show that up to 60% of adults suffering from mental illness report an inability to receive appropriate treatment and close to 80% seeking care prematurely discontinue psychotherapy [4-6]. Reasons for discontinuation include the stigma associated with psychotherapy, difficulties in connecting with therapists outside of clinical sessions, and poor fit with therapists [7,8].

In recent years, feedback-informed care (FIC) models have been used to improve patient compliance and psychotherapy outcomes [9,10]. FIC models are designed to encourage patients to provide feedback on care progress, assess well-being outside clinical sessions, and utilize a data driven approach to bolster the therapeutic alliance and build individually tailored therapies [9]. A key gap in FIC models has been the availability of resources and tools that allow patients to stay connected to their providers and capturing mental well-being data that currently can only be collected during relatively infrequent in-person psychotherapy sessions [11,12].

**Figure 1:**
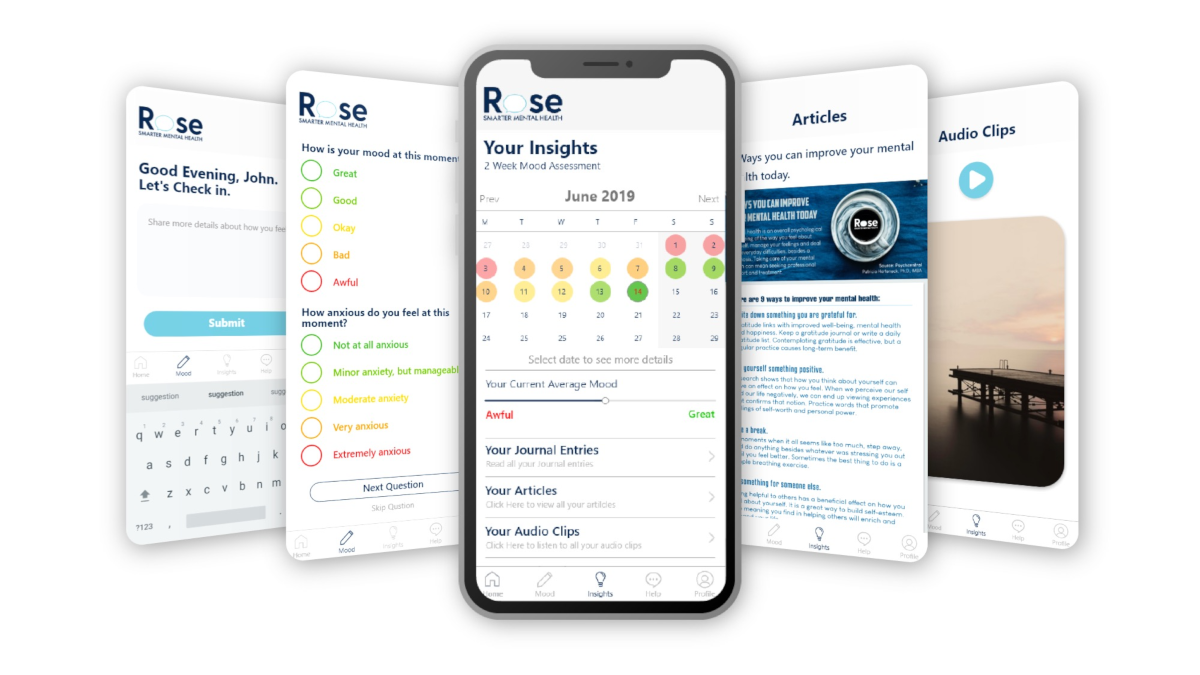
Rose mobile app screens.

The widespread use and availability of smartphones have made successful adoption of mobile technology increasingly crucial in developing FIC models [12-14] and identifying best practices. Unfortunately, of the 10,000 mental health apps currently available, less than 5% are backed by evidenced-based research [15]. Novel health technologies looking to revolutionize mental health care delivery and empower patients need to have an evidence-base that captures engagement of patients in their daily life with their therapists and the utility of collecting wellbeing data to address specific health concerns. The Rose digital health platform was developed in response to the clinical need for a robust tool to track mental wellbeing outside of clinical care. Rose’s digital health platform integrates a patient-facing mobile phone app for real-time mood tracking, clinical surveys, daily journaling, and curated insights with a web-based clinical dashboard allowing clinicians to check on their patient’s mental health status between clinical visits (Figure 1).

The primary goals of this feasibility study were to (1) assess the usage compliance with a mobile mental health platform among providers and their patients with at least mild-to-moderate depression and/or anxiety, and (2) examine the usability and fidelity of the mobile app through structured participant feedback. We hypothesized consistent usage adherence to the mobile platform over the study’s duration. A secondary exploratory aim evaluated the short-term impact of mobile app usage on mood and anxiety symptomatology.

## Methods

### Recruitment

Participants were recruited from the patient population at Key Point Health Services, a sizeable outpatient community psychiatric clinic in Baltimore, Maryland, USA. Inclusion criteria consisted of age 18 years or older, regularly making on-going appointments with a psychotherapist at the clinic, having a score of >5 on the Patient Health Questionnaire-9 (PHQ-9) and/or >5 on Generalized Anxiety Disorders-7 (GAD-7), having access to a smartphone, and being fluent in English. Exclusion criteria included a current diagnosis of decompensated psychosis (eg, hallucinations, delusions, thought disorder) and/or current suicidal or homicidal ideation. The cut-off of 5 on the PHQ-9 and GAD-7 represent mild depression and mild anxiety from clinical severity categorization [16,17]. Eligible patients were informed of the study via brochures provided by their established psychotherapists. Interested patients were directed to a secure online screener, which collected data on inclusion and exclusion criteria, demographics, and the PHQ-9 and GAD-7. This pilot was designed as an open-label trial with randomized allocation into study arms. A study member who was not involved in the day-to-day study operations used the STATA 14 (StataCorp) sequence generator feature for randomization. These were stored in sealed envelopes. At study registration and the online screening, the study coordinator opened the randomization envelopes to allocate the eligible participants to either the intervention arm or the control arm. Eligible, consented participants were then randomized via computer algorithm into one of two study arms: (1) the intervention arm or (2) the waitlist control arm, in a 2:1 ratio. All nine study therapists had appointments with at least one participant from each arm. Of note, no patient medical records were accessed as part of the study.

### Intervention Arm Design

Eligible participants continued meeting with their therapists for weekly psychotherapy sessions. Therapists were asked to register for the Rose web-based clinical dashboard, which generated a unique therapist ID. Participants in the intervention arm were asked to download the Rose app to their mobile device and were given their therapist’s unique ID for linking within the app. Participants were informed that the health metrics would be shared with their therapist to help the clinician better plan upcoming sessions. Participants were prompted to complete a prepilot battery of assessments (PHQ-9, GAD-7, mood and anxiety Likert scales) upon first logging into the app. This battery was also pushed to the users at 2- and 4-week (postpilot) time points. Those in the intervention arm were also asked to continue using the Rose app daily during the 4-week period. The therapist was asked to use the Rose clinical dashboard to review data in conjunction with each weekly in-clinic session for four sessions. All in-clinic sessions were scheduled in the same clinical space where participants previously received care. Each clinical session was approximately 45 minutes long, and therapist documentation was done using their standard of care electronic medical record system.

### Control Arm Design

The participants in the waitlist control arm served as controls for the study. They completed the prepilot and postpilot assessments (PHQ-9, GAD-7, mood and anxiety Likert scales) only. These participants continued their weekly standard care with the psychotherapy process. Waitlist participants were offered entrance to the intervention arm at the end of the study period or earlier if participants in the intervention arm dropped out mid-study. During their time on the waitlist, participants could reach out to study personnel if they needed assistance with their psychiatric care.

### Ethics

The study was reviewed and approved by the Advarra Commercial Institutional Review Board. For data security purposes, Rose utilizes cloud-based file-sharing and data storage services that are ISO 27001 certified and HIPAA (Health Insurance Portability and Accountability Act) compliant. If at any point during the study, participants denoted suicidal ideation or other acute psychiatric concerns, they were automatically provided with emergency contact numbers and the study clinician (AJ) reached out directly. Additionally, if a high-risk participant was identified during in-person psychotherapy sessions, the therapist contacted the study clinician immediately with any concerns. If it was deemed that a participant required a higher level of care (eg, inpatient hospitalization), they were removed from the study.

### Mobile Mental Health App (Rose)

Rose is a mobile health platform that consists of two primary components: (1) a patient-facing mobile app and (2) a clinician-facing web-based dashboard. Patients and clinicians are linked within the Rose system using a unique identifier that the clinician provides to the patient. The Rose app allows patients to track their mood and anxiety levels in real-time, complete validated assessments, and keep an in-app daily journal. Rose utilizes machine learning algorithms that identify mental health status and provide curated, individualized in-app daily insights for self-care. The Rose clinician-facing dashboard is designed for use in conjunction with in-person appointments. It is meant to augment in-person care by summarizing the data entered by the patient into the mobile app. Providers are given a walkthrough on using the dashboard and can reach the Rose team with any questions directly through the dashboard.

### Measures

The measures were as follows:

- Patient Health Questionnaire-9 (PHQ-9) [17] and Generalized Anxiety Disorder-7 (GAD-7) [18] Scale. The PHQ-9 and GAD-7 are valid and reliable screening tools for depression and anxiety, respectively. These assessments parallel the diagnostic symptom criteria that define DSM-IV major depression and GAD. The Rose app prompted users to complete these assessments every two weeks. The app follows the format and temporal framework corresponding to DSM-IV criteria.
- Modified Mobile Application Rating Scale (mMARS) (Multimedia Appendix 1) [19]. The MARS is a well-established framework for classifying and assessing the objective and subjective quality of apps, as well as their perceived impact. It is designed to score apps on the criteria of engagement, functionality, aesthetics, information quality, and subjective app quality. While the MARS framework is extensive, sections within the scale were not pertinent for this study and the overall length was deemed too tedious for patients to complete. The mMARS is a shortened version of the MARS, keeping the pertinent sections and modifying verbiage to reflect the current app usage.
- Mood & Anxiety Likert Scales [20]. The Rose app sends timed notifications once a day to rate current mood and anxiety, each on a 5-point Likert scale.
- In-app Journaling. The in-app journaling feature allows the user to enter a free text description of how they are doing, like in the case of a diary. A built-in sentiment analysis analyzes the entered sentences.

### Statistical Power Calculation

The power calculation for this study is based on a meta-analysis of internet-delivered treatments for adult depression and anxiety. It was found that internet interventions used in tandem with professional psychotherapy support showed an average between-group effect size of *d*=0.61 (large mean effect size of *d*=1.0). Based on the study’s five-psychotherapy session design (first session for on-boarding and four follow-up sessions), and paired-sample design (multiple measurements from the same person), a sample size of 30 has sufficient power (90%) to detect a moderately large effect size (*d*=0.8) [21]. Additionally, we recruited 15 patients as a waitlist control group who would be included in the intervention arm if drop-out or removal occurred.

### Primary Analyses

The primary analysis included checking distributional assumptions from the primary scores and assessing relationships among covariates. Continuous variables were described using mean, standard deviation, median, minimum and maximum, and 95% confidence interval for parameter estimation.

To determine whether any significant differences between groups existed at baseline, independent *t* tests were conducted on continuous baseline variables (eg, age, PHQ-9, GAD-7), and chi-square analyses were performed on categorical variables (gender, race, ethnicity).

Descriptive in-app metrics were calculated for compliance of assessments with PHQ-9 and GAD-7 every two weeks and daily mood/anxiety Likert scales and journaling. In-app use was also explored for time-of-day frequency and weekly variations.

- Adherence over time. Adherence was defined as the completion of in-app assessments at predefined intervals. For example, daily adherence to mood assessment was seen if a participant completed at least one of the mood or anxiety Likert scales each day. Non-adherence was recorded as days with no data. Each assessment measure was reviewed individually for the duration of the study (4 weeks) for the percentage of adherent days and was stratified for each study week in which the app was being used. Logistic regression models were carried out to examine whether adherence to in-app assessments would be associated with the severity of depressive symptoms at baseline.
- Fidelity of app use. Fidelity defines the degree to which programs are implemented as intended by the program developer [22]. In this study we looked at five main components of fidelity for the mobile app using the mMARS scale: Engagement, Functionality, Aesthetics, Quality of Information, and Subjective Quality. Summary proportions from the mMARS were reviewed in all five areas of app use.

### Exploratory Analyses

Pre-post analysis (Wilcoxon matched-pairs signed-rank test) was used to evaluate the short-term impacts of mobile app usage in the intervention arm and the control arm within in-person psychotherapy sessions.

All analyses were conducted using STATA 14 (StataCorp). All statistical tests were judged for significance based upon a two-tailed alpha level of *P*<.05. All subjects were included in intention-to-treat (ITT) analyses.

## Results

### Participant Characteristics

Table 1 shows the demographic information and baseline scores on clinical variables for those with data from the entire sample (n=45). Each therapist saw on average 3 patients (CI: 1-5). Participants in the intervention arm (n=30) were an average of 36 years old. Close to 60.00% of the participants were female and 29.03% reported completing college or higher level of education. Participants were mostly non-Hispanic making up 92.58%. 82.14% were Caucasian, 10.71% African American, and 7.14% identified with more than one race. Just over half (51.61%) used Android devices as their daily smartphone and the rest used Apple iOS devices. Other than the proportion of female participants (*P*=.02), there were no significant demographic differences between arms.

Looking at baseline mental health status, 42.22% of the sample was in the moderately-severe or severe range of depression, as measured by the PHQ-9. GAD-7 ratings showed 9.60% of the participants were in the moderately-severe or critical range for anxiety. There were no statistical differences in the distribution of participants randomized to each arm based on baseline mental health status.

**Table 1:** Demographic and baseline characteristics of study participants.

Characteristic		Total (n=45)	Intervention arm (n=30)	Waitlist arm (n=15)	*P* value
Age, median (range)		31 (18-65)	29 (18-65)	35 (19-54)	.75
Female sex, n (%)		29 (70.73%)	16 (60.71)	12 (92.31)	.02
**Education, n (%)**					.32
	High school or less	29 (64.44)	19 (63.33)	10 (76.92)	
	College or higher	12 (26.67)	9 (30.00)	29.03%	
**Race, n (%)**					.74
	White	33 (78.57%)	25 (83.33)	9 (69.23)	
	Black	5 (11.90%)	3 (10.71)	2 (15.38)	
	Hispanic	1 (2.38%)	0 (0)	1 (7.69)	
	Mixed	3 (7.14%)	2 (7.14)	1 (7.69)	
**Device, n (%)**					.62
	Android	19 (42.22%)	14 (46.67)	5 (38.46)	
	Apple iOS	25 (55.56%)	16 (53.33)	8 (61.54)	
PHQ-9, mean (SD)		13.47 (5.90)	12.35 (5.57)	16.08 (5.32)	.89
GAD-7, mean (SD)		11.96 (6.02)	11.19 (5.77)	13.23 (6.50)	.74

### Adherence Over Time

Participants in the intervention arm used the Rose mobile app over 4 weeks as part of care management. Table 2 details the descriptive statistics on participants’ daily use of the mood Likert scale, anxiety Likert scale, and online journaling; overall and by study week. Over the study period, patients adhered to completing all three assessments at an average of 69% per week (5 days a week). As seen in Table 2, adherence across all three estimates was reasonably similar. Adherence was highest in the first week, averaging at 81.17% (95% CI: 75.31%-87.04%) across all three assessments, with a linear decrease at week 4 to 56.03% (95% CI: 43.92%-68.14%) adherence. Majority adherence of 80% (24/30 days) was seen in 14 of 30 participants completing the mood Likert scale, 15 of 30 participants completing the anxiety Likert scale, and 13 of 30 participants completing the journal.

Looking at the use of PHQ-9 and GAD-7 in-app scales (Table 3), participants adhered to filling in all three assessments at an average rate of 97% over the study period (every two weeks). Full adherence (3/3 assessments) was seen in 27/30 of the patients.

**Table 2:** Weekly adherence rates for in-app mood and anxiety Likert scales and journaling.

Week	Adherence % (SD)		
	Mood check-in	Anxiety check-in	Journal
1	82.86 (14.24)	81.90 (16.06)	76.19 (18.89)
2	70.95 (25.02)	70.48 (28.13)	60.95 (33.19)
3	64.29 (29.95)	61.90 (32.10)	52.86 (33.91)
4	58.10 (32.69)	62.86 (33.79)	57.14 (35.04)

**Table 3:** Biweekly adherence rates for in-app PHQ-9 and GAD-7 assessments.

Week	Adherence % (SD)	
	PHQ-9^a^	GAD-7^b^
0	100 (0)	100 (0)
2	100 (0)	100 (0)
4	90 (30.51)	90 (30.51)

^a^PHQ-9: Patient Health Questionnaire 9.

^b^GAD-7: Generalized Anxiety Disorder 7.

### Fidelity of App Use

**Figure 2:**
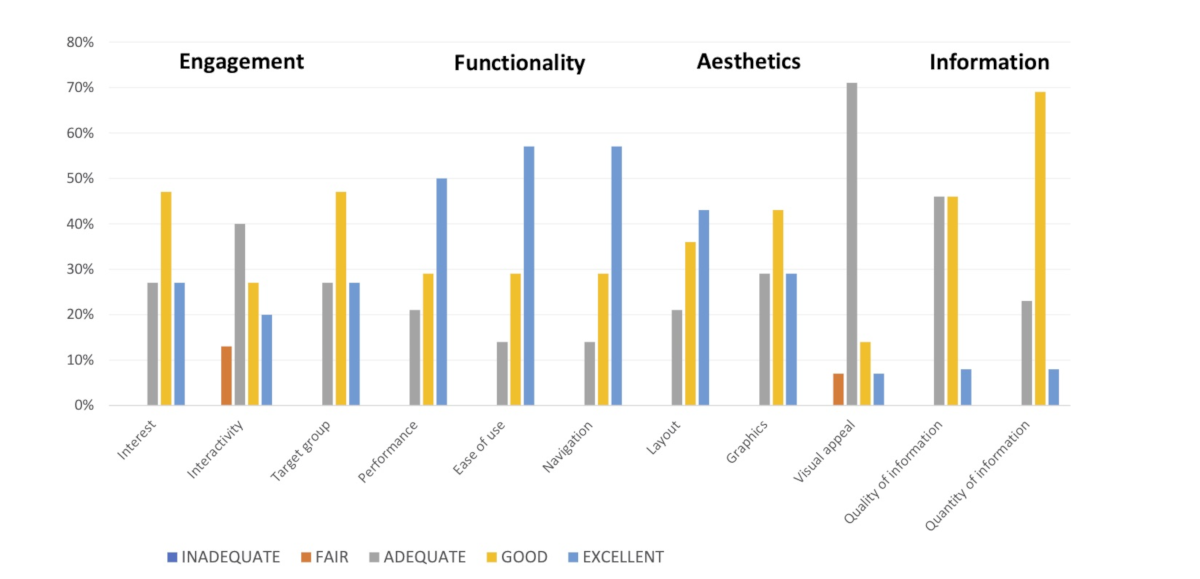
mMARS – Perceived Usability of Rose.

Using the mMARS scale we evaluated the perceived usability of the mobile app as rated by participants (Figure 2). Overall, participants thought the app was very engaging, with approximately 73% finding Rose very interesting and appropriate for the target audience. Approximately 83% of the participants reported the app to be very accurate, easy to use, and easy to navigate. Similarly, 75% reported the layout and graphics to be very pleasing and 71% felt the overall look was only adequate. Furthermore, 54% reported that the information provided through the app was of good or excellent quality, and 77% of the participants felt it covered a comprehensive range of topics. Overall, 67% said they would recommend the app outside of the study.

**Figure 3:**
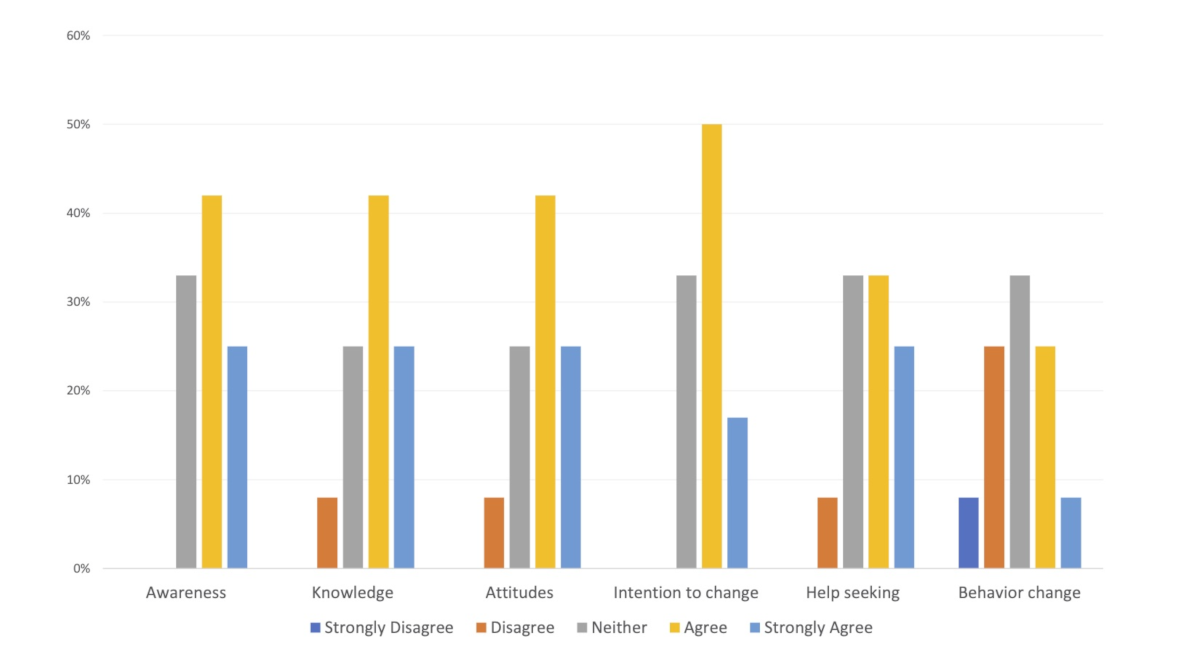
Impact of App Usage on Mental Health Knowledge and Attitudes.

We further asked participants about the perceived impact of the Rose app on their knowledge, attitudes, intentions to change, as well as the likelihood of actual change in depression and/or anxiety symptoms (Figure 3). More than two-thirds of the participants felt that their knowledge of the topic and awareness of the importance of depression and anxiety management improved through using the app. Similarly, 67% reported they were more than likely to take progressive steps to address depression and more than half stated that the app made them likely to seek help for depressive symptoms when needed.

### Exploratory Analyses

We looked for changes in depression and anxiety symptoms (measured through PHQ-9 and GAD-7) before and after the study in both the intervention and control arms (Table 4).

Participants in the intervention arm showed statistically significant improvements in measurements of both depression and anxiety symptoms. On the PHQ-9, there was an average 4-point improvement in participants over four weeks (prepilot average score: 15 vs postpilot average score: 11; *P*=.01). On the GAD-7, there was an average 3-point improvement in participants over four weeks (prepilot average score: 11 vs postpilot average score: 8; *P*=.005). Overall, 47% of patients with moderate-to-severe depression improved to mild depression and 56% of patients with moderate-to-severe anxiety improved to mild anxiety by the end of the study. Additionally, Kruskal-Wallis H test revealed statistically significant improvements in both PHQ-9 (*P*=.02) and GAD-7 (*P*=.001) scores over the course of the study in the intervention group.

Among the six controls who completed the pre- and postpilot assessments, neither PHQ-9 nor GAD-7 scores showed any significant changes (Table 5). Even though there was a difference in mean scores on both PHQ-9 and GAD-7 scales, statistical comparison on PHQ-9 and GAD-7 scores between intervention and control arms did not show statistical significance.

**Table 4:** Pre-post change in depression and anxiety symptoms: intervention (n=30) vs control (n=15).

Variable		Prepilot assessment, mean (SD)	Postpilot assessment, mean (SD)	*P* value
**Intervention arm (n=30)**				
	PHQ-9^a^	15.33 (8.94)	11.04 (8.35)	.01
	GAD-7^b^	11.43 (6.46)	8.17 (6.06)	.005
**Control arm (n=15)**				
	PHQ-9	13.83 (6.74)	13.17 (7.68)	.53
	GAD-7	10.17 (7.86)	10.17 (8.75)	1.0

^a^PHQ-9: Patient Health Questionnaire-9.

^b^GAD-7: Generalized Anxiety Disorder-7.

**Table 5:** Pre-post analysis of changes in depression and anxiety symptoms.

Variable	Intervention arm, mean (SD)	Control arm, mean (SD)	*P* value
PHQ-9^a^ change	–4.30 (1.54)	0.67 (0.99)	.11
GAD-7^b^ change	–3.11 (1.23)	0 (1.29)	.41

^a^PHQ-9: Patient Health Questionnaire-9.

^b^GAD-7: Generalized Anxiety Disorder-7.

## Discussion

### Principal Findings

This study demonstrated the fidelity of using a digital mental health platform (Rose) for monitoring daily mental health and wellbeing outside of psychotherapy sessions and maintaining engagement in patients with depression and anxiety symptoms. Exploratory analyses examined changes in depressive and anxiety symptoms at baseline versus after study interventions. Improved PHQ-9 scores were observed in 76% (23/30) of intervention arm participants and 70% (21/30) showed improvements in their GAD-7 scores over the study period. Furthermore, patients rated the Rose mobile app as having high engagement and usability as well as content curation relevant to their needs.

The study paralleled current psychotherapy care practices for moderate depression. These results showed using Rose is no worse than not using an app within psychotherapy sessions. Consistent compliance by patients between and during clinical psychotherapy sessions indicates that high levels of adherence can be achieved and retained. A larger multisite randomized controlled trial is being planned to look at the impact of the Rose digital health platform with an active (alternative mobile app) control.

### Comparison With Prior Work

The economic impact of mental illness is marked, with the United States alone seeing $210 billion in annual medical expenditures tied to mental health disorders [23,24]. The added societal burdens of mental illness include productivity losses, absenteeism, and reduced economic growth [25]. On average, depressive symptoms lead to approximately 27 lost workdays and 18 days of reduced productivity per year in people diagnosed with depression [26]. Research attention to address these needs has exponentially increased in recent years, especially around the area of mobile mental health apps [14,27]. Research groups working in this space have found, through both observational and clinical trials, positive impacts on the utility of smartphones with onboard sensors to diagnose psychiatric disorders [12]. However, studies of adherence to use of mobile mental health apps have been limited. A meta-analysis by Tourous et al looked at 18 clinical trials that investigated the efficacy of smartphone interventions targeting depressive symptoms. The authors note that lower rates of engagement over time have been found in numerous mental health app studies [28], with higher rates being more common in shorter intervention interactions. Rates of adherence were notably higher with apps that had added components of human support, with real world engagement rates close 17% for peer support apps, but adding human interactions may reduce scalability, especially in low resource settings [28]. This suggests that intervention designs have to be adaptable and customizable to the ways in which people use smartphones. Results from the current study demonstrate the utility of smartphone-driven interventions to improve on FIC and in maintaining nonhuman-supported adherence. Furthermore, there are few studies that have looked at design, fidelity, and usability of mental health apps tied to intervention outcome and patient engagement [29,30]. This study is also one of the first to look at mobile mental health app outcome metrics and interaction principles in a longitudinal investigational format.

### Limitations

Participants in this study were selected from a single study site and were not randomized by therapists. Thus, there may be a sampling bias that limits generalizability of the results. Furthermore, the sample consisted of 60%-90% women, which is not representative of the population of individuals with depression/anxiety in clinical settings or the community. While the pilot study had a waitlist control arm, it did not include an active comparison group (ie, using an alternate mobile app). Furthermore, only 40% of the control arm completed the postpilot follow-up. This limited the pre-post analytical comparison. A follow-up randomized control trial is needed to evaluate inferences regarding the efficacy and contrast of effect sizes between the Rose digital health platform and other interventions.

### Conclusions

Steady compliance and high app ratings showcase the usability of Rose in practice and the utility of digital FIC models in improving patient compliance to therapy. Although exploratory, reduction in clinical depression and anxiety symptoms over the four-week study period highlights the potential utility of the Rose app for psychotherapy augmentation. Future studies are being developed to (1) further evaluate the clinical effectiveness of the Rose app through a multisite micro-randomized clinical trial, and (2) examine the impact of the Rose digital health platform on other diagnoses, including substance use and pain.

## Appendix

Multimedia Appendix 1 Modified Mobile Application Rating Scale (mMARS).

## Abbreviations

FIC: feedback-informed care
GAD-7: Generalized Anxiety Disorder-7
HIPPA: Health Insurance Portability and Accountability Act
ITT: intention-to-treat (ITT)
mMARS: modified Mobile Application Rating Scale
PHQ-9: Patient Health Questionnaire-9
