# Supplementary material for: Use of a Mobile App to Augment Psychotherapy in a Community Psychiatric Clinic: Feasibility and Fidelity Trial
Source: JMIR Form Res. 2020 Jul 3;4(7):e17722. doi: 10.2196/17722 (PMC7367543; doi:10.2196/17722)
Supplement: Multimedia Appendix 1 [file formative_v4i7e17722_app1.pdf]

INTERVENTION ARM SURVEY

Congratulations on completing the study!

We would like you to complete a few final surveys.

This does not impact your on-going clinical care at all.

You are welcome to continue using the Rose application with your provider.

INTERVENTION ARM SURVEY

APP QUALITY RATINGS: The rating scale assesses app quality on four dimensions.

SECTION A: Engagement – fun, interesting, customizable, interactive (e.g. sends alerts, messages, reminders, feedback, enables sharing), well-targeted to audience

1. Interest: Is the app interesting to use? Does it present your health status and surveys in an interesting way?

- ○ 1. Not interesting at all
- ○ 2. Mostly uninteresting
- ○ 3. Ok, neither interesting nor uninteresting
- ○ 4. Moderately interesting
- ○ 5. Very interesting

2. Interactivity: Does it allow user input, provide feedback, contain prompts (reminders, sharing options, notifications, etc.)?

- ○ 1. No interactive features and/or no response to user interaction
- ○ 2. Insufficient interactivity, or feedback, or user input options, limiting functions
- ○ 3. Basic interactive features to function adequately
- ○ 4. Offers a variety of interactive features/feedback/user input options
- ○ 5. Very high level of responsiveness through interactive features/feedback/user input options

INTERVENTION ARM SURVEY

3. Target group: Is the app content (visual information, language, design) appropriate for you

- ◯ 1. Completely inappropriate/unclear/confusing
- ◯ 2. Mostly inappropriate/unclear/confusing
- ◯ 3. Acceptable but not targeted. May be inappropriate/unclear/confusing
- ◯ 4. Well-targeted, with negligible issues
- ◯ 5. Perfectly targeted, no issues found

SECTION B: Functionality – app functioning, easy to learn, navigation, flow logic, and gestural design of app.

4. Performance: How accurately/fast do the app features (functions) and components (buttons/menus) work?

- ◯ 1. App is broken; no/insufficient/inaccurate response (e.g. crashes/bugs/broken features, etc.)
- ◯ 2. Some functions work, but lagging or contains major technical problems
- ◯ 3. App works overall. Some technical problems need fixing/Slow at times
- ◯ 4. Mostly functional with minor/negligible problems
- ◯ 5. Perfect/timely response; no technical bugs found/contains a 'loading time left' indicator

INTERVENTION ARM SURVEY

5. Ease of use: How easy is it to learn how to use the app; how clear are the menu labels/icons and instructions?

- ◯ 1. No/limited instructions; menu labels/icons are confusing; complicated
- ◯ 2. Useable after a lot of time/effort
- ◯ 3. Useable after some time/effort
- ◯ 4. Easy to learn how to use the app (or has clear instructions)
- ◯ 5. Able to use app immediately; intuitive; simple

6. Navigation: Is moving between screens logical/accurate/appropriate/ uninterrupted; are all necessary screen links present?

- ◯ 1. Different sections within the app seem logically disconnected and random/confusing/navigation is difficult
- ◯ 2. Usable after a lot of time/effort
- ◯ 3. Useable after some time/effort
- ◯ 4. Easy to use or missing a negligible link
- ◯ 5. Perfectly logical, easy, clear and intuitive screen flow throughout, or offers shortcuts

INTERVENTION ARM SURVEY

SECTION C: Aesthetics – graphic design, overall visual appeal, colour scheme, and stylistic consistency

7. Layout: Is arrangement and size of buttons/icons/menus/content on the screen appropriate or zoomable if needed?

- ◯ 1. Very bad design, cluttered
- ◯ 2. Bad design, random, unclear, some options difficult to select/locate/see/read
- ◯ 3. Satisfactory, few problems
- ◯ 4. Mostly clear, able to select/locate/see/read items
- ◯ 5. Professional, simple, clear, orderly

8. Graphics: How high is the quality/resolution of graphics used for buttons/icons/menus/content?

- ◯ 1. Graphics appear amateur, very poor visual design
- ◯ 2. Low quality/low resolution graphics; low quality visual design
- ◯ 3. Moderate quality graphics and visual design
- ◯ 4. High quality/resolution graphics and visual design –
- ◯ 5. Very high quality/resolution graphics and visual design

INTERVENTION ARM SURVEY

9. Visual appeal: How good does the app look?

- 1. No visual appeal
- 2. Little visual appeal
- 3. Some visual appeal
- 4. High level of visual appeal
- 5. As above + very attractive, memorable, stands out

SECTION D: Information – Contains high quality information (e.g. text, feedback, measures, references) from a credible source. Select N/A if the app component is irrelevant.

10. Quality of information: Is app content correct, well written, and relevant to the goal/topic of the app?

- 1. Irrelevant/inappropriate/incoherent/incorrect
- 2. Poor. Barely relevant/appropriate/coherent/may be incorrect
- 3. Moderately relevant/appropriate/coherent/and appears correct
- 4. Relevant/appropriate/coherent/correct
- 5. Highly relevant, appropriate, coherent, and correct

INTERVENTION ARM SURVEY

11. Quantity of information: Is the extent coverage within the scope of the app; and comprehensive but concise?

- ◯ 1. Minimal or overwhelming
- ◯ 2. Insufficient or possibly overwhelming
- ◯ 3. OK but not comprehensive or concise
- ◯ 4. Offers a broad range of information, has some gaps or unnecessary detail; or has no links to more information and resources
- ◯ 5. Comprehensive and concise; contains links to more information and resources

What did you think of Rose in general

12. Would you recommend this app to people who might benefit from it?

- ◯ 1. Not at all - I would not recommend this app to anyone
- ◯ 2. There are very few people I would recommend this app to
- ◯ 3. Maybe - There are several people whom I would recommend it to
- ◯ 4. There are many people I would recommend it to
- ◯ 5. Definitely - I would recommend this app to everyone

INTERVENTION ARM SURVEY

App-specific Impact

	Strongly Disagree	Disagree	Neither Agree Nor Disagree	Agree	Strongly Agree
This app is likely to increase awareness of addressing mental health	○	○	○	○	○
This app is likely to increase knowledge/understanding of mental health	○	○	○	○	○
This app is likely to change attitudes towards improving mental health	○	○	○	○	○
This app is likely to increase intentions/motivation to address mental health	○	○	○	○	○
Use of this app is likely to encourage further help seeking for mental health	○	○	○	○	○
Use of this app is likely to increase mental health	○	○	○	○	○

INTERVENTION ARM SURVEY

Three things you liked about Rose

1.
2.
3.

Three things you didnt like about Rose

1.
2.
3.

Would you continue using Rose as part of your therapy?

- ◯ Yes
- ◯ Maybe
- ◯ No

INTERVENTION ARM SURVEY

14. Would you pay for this app?

- 1. No
- 3. Maybe
- 5. Yes

14. What is your overall star rating of the app?

- 1. One Star - One of the worst apps I've Used
- 2. Two Stars
- 3. Three Stars - Average
- 4. Four Stars
- 5. Five Stars - One of the best apps I've used

INTERVENTION ARM SURVEY

Would you be willing to speak to us about your time using Rose?

- ◯ Yes
- ◯ No

Would you be willing to be contacted in the future as a follow-up to this study?

- ◯ Yes
- ◯ No
